# Tunable Control of Interlayer Excitons in WS_2_/MoS_2_ Heterostructures via Strong Coupling with Enhanced Mie Resonances

**DOI:** 10.1002/advs.201802092

**Published:** 2019-04-02

**Authors:** Jiahao Yan, Churong Ma, Yingcong Huang, Guowei Yang

**Affiliations:** ^1^ State Key Laboratory of Optoelectronic Materials and Technologies Nanotechnology Research Center School of Materials Science & Engineering Sun Yat‐sen University Guangdong Guangzhou 510275 P. R. China

**Keywords:** heterostructures, MoS_2_, silicon nanoparticles, tunable interlayer exciton, WS_2_

## Abstract

Strong Coulomb interactions in monolayer transition metal dichalcogenides (TMDs) produce strongly bound excitons, trions, and biexcitons. The existence of multiexcitonic states has drawn tremendous attention because of its promising applications in quantum information. Combining different monolayer TMDs into van der Waals (vdW) heterostructures opens up opportunities to engineer exciton devices and bring new phenomena. Spatially separated electrons and holes in different layers produce interlayer excitons. Although much progress has been made on excitons in single layers, how interlayer excitons contribute the photoluminescence emission and how to tailor the interlayer exciton emission have not been well understood. Here, room temperature strong coupling between interlayer excitons in the WS_2_/MoS_2_ vdW heterostructure and cavity‐enhanced Mie resonances in individual silicon nanoparticles (Si NPs) are demonstrated. The heterostructures are inserted into a Si film‐Si NP all‐dielectric platform to realize effective energy exchanges and Rabi oscillations. Besides mode splitting in scattering, tunable interlayer excitonic emission is also observed. The results make it possible to design TMDs heterostructures with various excitonic states for future photonics devices.

## 1 Introduction

Transition metal dichalcogenides (TMD) monolayers have attracted great attention for intriguing optical and electronic properties. The direct bandgap and high quantum yield in TMDs monolayer generate strong photoluminescence (PL) emission.^1^, ^2^ Strong excitonic effects and larger exciton binding energies have also been reported for TMDs.^3^, ^4^, ^5^ Due to the strong Coulomb interactions, trions and biexcitons with three and four charge carriers are also generated together with excitons.^6^, ^7^ It has been reported the excitonic states can be modulated via chemical doping,^3^ gate voltages,^5^ or strains.^8^ Additionally, ultrathin 2D materials are also unique and effective platforms to build heterostructures via van der Waals (vdW) forces.^9^, ^10^ The optoelectronic properties and excitonic states of heterostructures can be engineered through different TMDs^11^, ^12^ and different stacking orientation.^13^, ^14^ In type‐II band alignment vdW heterostructures, efficient charge transfer, and separation in different layers can occur and produce interlayer excitons with long lifetimes and valley depolarization lifetimes.^9^, ^12^, ^15^ Interlayer excitons in WS_2_/MoS_2_ heterostructures have been reported located between the excitonic transition energies of WS_2_ and MoS_2_ 16, 17 or lower than them.^10^, ^18^ Furthermore, the interlayer coupling between WS_2_ and MoS_2_ strongly depends on the stacking orientation.^13^ No exact location of interlayer exciton in WS_2_/MoS_2_ has been established. How to modulate the interlayer excitons also needs to figure out.

For monolayer TMDs, plasmonic nanostructures^19^, ^20^ and photonic crystals^21^, ^22^ have been utilized to realize strong photon–exciton interaction. Optical resonant cavities can change the emission lifetime of embedded excitonic dipoles through Purcell effect.^23^, ^24^ When the coupling strength is strong enough, fast energy exchange between exciton and cavity generates Rabi oscillations and forms the cavity polaritions.^23^, ^24^, ^25^ The strong coupling leads to complex and hybrid exciton emission. However, there are very few works on the strong coupling between interlayer excitons in vdW heterostructures and optical cavities. Therefore, new platform needs to be found to integrate with vdW heterostructures.

Here we first demonstrate cavity‐enhanced Mie resonators as an effective platform for strong coupling with interlayer excitons in TMDs heterostructures at room temperature. This all‐dielectric platform consists of a Si NP and a subwavelength Si film separated by a thin SiO_2_ layer. Compared with widely used Mie resonators,^26^, ^27^ Si NPs supported on a nanocavity obtain much higher field enhancement. Furthermore, regular Mie resonances dominated by field enhancements inside nanoparticles transform to a quasi‐surface mode possessing strong field enhancements on surfaces and interfaces. This feature is favorable to obtain large coupling strength.^28^ On the other hand, interlayer excitons possess an out‐of‐plane dipole component rather than usual in‐plane dipoles in monolayer TMDs, which makes it more efficiently to couple to localized electric fields.^15^, ^29^ Kleemann et al. has demonstrated the strong coupling between multilayer TMDs and plasmonic nanocavities because exciton dipole in multilayers has 25% out‐of‐plane component.^30^ In the proposed interlayer exciton‐Mie resonator strong coupling system, we observed the red‐shift or blue‐shift of interlayer excitonic emission for the first time. The degree of shift depends on the sizes of Si NPs. The maximum red shift can reach 0.03 eV in random stack heterostructures, while the maximum blue shift we observed is 0.05 eV in coherent stack heterostructures. In addition, anticrossing behaviors are observed in dark‐field scattering spectra to further demonstrate the strong coupling. To our knowledge, this is the strongest coupling based on Si NPs.^31^, ^32^ These findings present a new method on the control of excitonic effects which is crucial to build excitonic devices in the future.

## 2 Results and Discussion

**Figure 1:**
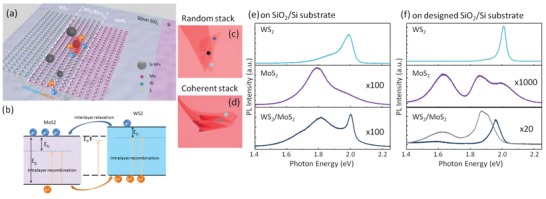
Interlayer excitons in the WS_2_/MoS_2_ van der Waals heterostructure. a) Schematic depiction of Si NPs decorated WS_2_/MoS_2_ heterostructure on the designed SiO_2_/Si substrate. b) Band alignment in the heterostructure with the generation of intralayer and interlayer excitons. Purple and blue colors represent bands in MoS_2_ and WS_2_ respectively. *E*_g_ and *E*_b_ mean the energy gap and exciton binding energy respectively. c) Optical images of two types heterostructures. f) Dots with different colors correspond to spectra with different colors. d) PL spectra collected from a WS_2_ monolayer (blue curve), a MoS_2_ monolayer (purple curve), and the WS_2_/MoS_2_ area (black curve) on conventional SiO_2_/Si substrate. The spectra are scaled by labeled amplification for visual convenience. e) PL spectra of WS_2_ monolayer (blue curve), MoS_2_ monolayer (purple curve), the random‐stacking WS_2_/MoS_2_ (black curve), and the coherent‐stacking WS_2_/MoS_2_ (gray curve) on designed SiO_2_/Si substrate.

WS_2_ and MoS_2_ monolayers were chosen to form vdW heterostructures. MoS_2_ monolayer is transferred to the designed SiO_2_/Si substrate (50 nm SiO_2_+200 nm Si+375 nm SiO_2_+back Si), followed by dry transfer^33^ of WS_2_ on it. Si NPs with different sizes from 80 to 200 nm were synthesized and deposited on heterostructures as illustrated in **Figure** **1**a. Excitonic energies of WS_2_, MoS_2_, and WS_2_/MoS_2_ perfectly match the resonant energies of our fabricated Si NPs. Under excitation at 514 nm, interlayer excitons might be formed, and the electrons and holes can be immediately distributed on energetically preferred states in MoS_2_ and WS_2_ respectively. As shown in Figure 1b, multiple excitonic states exist in type‐II band alignment WS_2_/MoS_2_ heterostructures such as excitons (X_0_), trions (X^−^) from intralayer recombination and the interlayer excitons (X_i_) from interlayer relaxation. The type‐II alignment can narrow the bandgap and produce interlayer excitons around 1.4 eV.^10^, ^15^ However, some works also demonstrated the interlayer exciton energy is located between the excitonic energies of WS_2_ and MoS_2_.^16^, ^34^ Since the binding energy of interlayer exciton is much lower, the interlayer excitonic emission may become larger than that of MoS_2_ but smaller than WS_2_.^16^ Two typical heterostructures are presented in Figure 1c,d. The assembled processes in Figures S1 and S2 (Supporting Information) indicate the shape of MoS_2_ and WS_2_ layers. The number of layers can be determined through optical contrast, which is similar to conventional SiO_2_/Si substrates. Bright‐field and dark‐field optical images reveal the distribution of Si NPs.

Figure 1e,f are the PL spectra before depositing Si NPs. For monolayer TMDs on the SiO_2_ (300 nm)/Si substrate, a strong emission peak can be observed at 1.98 eV for monolayer WS_2_ and a emission peak at 1.78 eV accompany with a weak B excitonic emission around 2.00 eV is observed in monolayer MoS_2_.^3^ Those line shapes are in accordance with most reported works.^35^, ^36^ After forming as heterostructures, the PL intensities experience two orders of magnitude decrease compared with that of monolayer WS_2_. The line shape is the superposition of PL line shapes of WS_2_ and MoS_2_ monolayers in general, and no interlayer excitonic emission peak is observed. PL spectra of TMDs monolayer strongly depend on the substrate and optical resonant modes.^37^ Similarly, besides clean interface and good contact, types of substrate also play important role on the interlayer coupling.^17^ In our case, the Fabry–Perot mode in the designed SiO_2_/Si substrate makes the PL line shapes of WS_2_ and MoS_2_ different from those on the SiO_2_ (300 nm)/Si substrate. For the individual WS_2_ monolayer, stronger and narrower excitonic emission peak is obtained at 2.01 eV. For MoS_2_ monolayer, besides the A and B excitons located at 1.86 and 1.99 eV respectively, a strong emission peak arising from the cavity mode is also generated at 1.63 eV. Series of vdW heterostructures on the designed SiO_2_/Si substrate were fabricated. Behaviors of interlayer exciton depend on the stacking orientation. In general, there are two states: “random stack” and “coherent stack.”^13^ Whether the vdW heterostructure is “random stack” or “coherent stack” depends on the stacking angle. “Coherent stack” means no rotation misfit between the hexagon unit‐cell of MoS_2_ and WS_2_. The stacking angle greatly influences the charge‐transfer dynamics which determines the interlayer exciton population and energy.^13^ On the other hand, the interlayer coupling strength is weaker at “random stack” owing to the highly mismatch atomic alignment, indicating that the interlayer distance is larger.^14^ Figure 1f presents the PL spectra of two states marked in Figure 1c,d. The interlayer excitonic peaks both locate between the peaks from pure WS_2_ and MoS_2_. The emission peak of random‐stack heterostructure locates at 1.96 eV which has a 0.05 eV red shift from the excitonic peak of WS_2_ (2.01 eV), while the excitonic peak of coherent‐stack heterostructure has a blue shift compared with that of MoS_2_. Besides, the peaks at 1.88 and 1.62 eV both experience shifts compared with individual MoS_2_ monolayers, which further demonstrate the existence of interlayer coupling. As talked above, the excitonic peak located between the peaks from pure WS_2_ and MoS_2_ does indicate the strong interlayer coupling. Tongay et al.^34^ has reported a new PL peak in the WS_2_/MoS_2_ heterostructure named P_hetero_ whose energy is 0.06 eV below P_WS2_. Zhang et al.^16^ also observed interlayer excitons at 1.94 eV. Although excitonic emission from TMDs monolayer may experience blue‐shift when supported by other 2D materials,^38^ the PL spectrum in Figure 1e indicates the excitonic peak of WS_2_ has no shift with the existence of MoS_2_. Therefore, we can exclude the dielectric screening affect on the PL shift.

**Figure 2:**
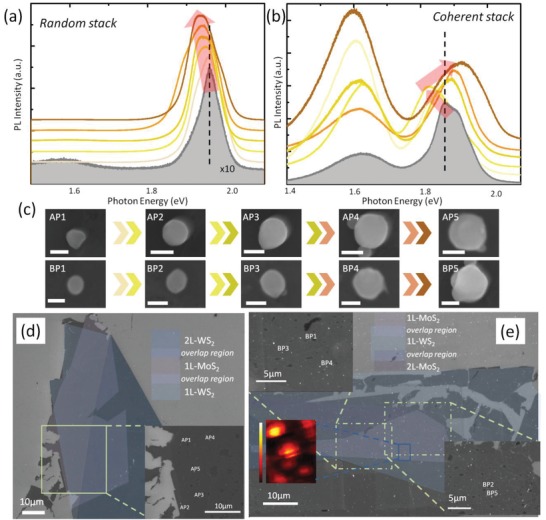
PL spectra of WS_2_/MoS_2_ heterostructures with Si NPs. Tunable PL emission of the a) random‐stacking and b) coherent‐stacking heterostructures with different Si NPs on it. Filled spectrum is the PL emission without Si NPs. Curves with darker colors means Si NPs with larger sizes. The red arrow indicates the spectral variation trend. c) SEM images showing measured Si NPs deposited on random‐stacking (AP1–AP5) and coherent‐stacking (BP1–BP5) heterostructures. Arrows mean the trend of increase, and all scale bars are set to 100 nm. d,e) SEM images with different magnifications labeling the locations of measured spots. Different TMD layers are labeled by semi‐transparent polygons with different colors. Inset in (e) is the typical PL intensity mapping.

After depositing Si NPs on heterostructures, the excitonic emission can be further modulated. In **Figure** **2**a, we measured the PL spectra at different locations of random‐stack sample with different Si NPs on. The interlayer excitonic energy experiences significant red‐shift from 1.96 to 1.93 eV with the increase of particle size from 100 to 160 nm. Si NPs we measured are presented in Figure 2c. Although the nanoparticles are not uniform circular shape, the imperfect shape and adherent small particles have little influence on the optical properties and resonant modes as we reported before.^26^ Contradictory trend was observed in coherent‐stack heterostructures. When increasing the sizes of Si NPs, the interlayer excitonic emission first red‐shifts and then strongly blue‐shifts (up to 0.05 eV) as shown in Figure 2b. Figure 2c shows the shapes of Si NPs measured in Figure 2b with sizes from 100 to 200 nm (BP1, BP2–BP5). Figure 2d,e with insets is the scanning electron microscope (SEM) images with different magnifications to reveal the locations of different Si NPs. Si NPs synthesized from laser ablation method have a wide size distribution which is beneficial for us to do a series of measurements in one sample. However, the random deposition makes irregular spatial distribution and size distribution. Therefore, wanted NPs must be found in a large area. In Figure 2e, PL mapping data is also attached to demonstrate the enhancement of interlayer emission caused by Si NPs. Although interlayer excitonic emission has much lower intensity than pure monolayer TMDs due to interlayer relaxation,^17^ the existence of Si NPs can reboost the emission intensity with more than ten times enhancement factor.

**Figure 3:**
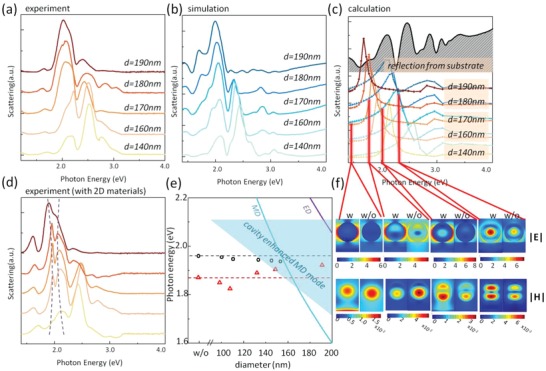
Analysis on the strong coupling between interlayer excitons and enhanced Mie resonances. a) Measured scattering spectra of Si NPs on cavity with different diameters (140, 160, 170, 180, and 190 nm). b) Simulated scattering spectra of Si NPs on cavity with different diameters. c) Calculated scattering contributed by ED and MD modes with the simulated reflection from designed SiO_2_/Si substrate. ED modes are plotted using series of cool colors, while MD modes are plotted using warm colors. d) Measured scattering spectra of Si NPs with different diameters deposited on heterostructures. Dash lines show the anti‐crossing behavior. e) Diameter dependence of the energies of interlayer excitons. The locations of MD and ED modes, and the range of cavity enhanced MD mode. f) Electric (|*E*|) and magnetic (|*H*|) field distributions of Si NPs on cavity (labeled by “w”) and on pure oxide (labeled by “w/o”) at four different locations in spectrum marked by red bars.

To explain how Si NPs affect the excitonic behaviors, optical properties of Si NPs should be studied. It should be noticed that the resonant mode of Si NPs in our case is different from normal Mie resonators^26^, ^27^ with a magnetic dipole (MD) resonant mode and an electric dipole (ED) resonant mode. The special substrate brings additional cavity modes effectively coupling with Mie resonators. **Figure** **3**a is the measured dark‐field scattering spectra of Si NPs on cavity with different sizes. No obvious red‐shift is observed when increasing sizes, but the drop‐off of scattering peak around 2.50 eV and the enhancement of the peak around 2.10 eV can be seen clearly. The trend is verified in simulation as shown in Figure 3b. Spectral fine structure can be seen in simulation much easier because perfect nanosphere and ideal surroundings were used in simulation. The reason why 50 nm top oxide layer was used in the designed SiO_2_/Si substrate is because NPs on the 50 nm oxide layer have the strongest field enhancement and the most suitable spectral line shape. A series of scattering spectra and field profiles of Si NPs on oxide films with different thickness are presented in Figure S3 in the Supporting Information. Mie theory is utilized to analysis the scattering contributed by MD and ED modes respectively. In Figure 3c, besides the MD and ED responses of Si NPs with different sizes, reflection from the designed substrate is also presented to reveal how cavity modes affect the Mie resonances. For Si NPs with diameters from 140 to 190 nm, the MD mode shifts linearly from 2.13 to 1.62 eV. Similarly, the ED mode moves from 2.58 to 1.95 eV linearly. While the reflection peaks at 2.08 and 2.46 eV modulate the Mie resonant modes and produce cavity‐enhanced MD and ED modes. The cavity‐enhanced modes are more favorable to realize strong coupling with ultrathin TMDs. Figure 3d is the measured scattering spectra of Si NPs on WS_2_/MoS_2_ heterostructures. The modified Mie resonant mode is located in the range from 1.90 to 2.13 eV, which is perfectly overlapped with the excitonic energy in scattering spectra at 2.00 eV. Therefore, significant mode splitting with the high‐ and low‐energy hybrid modes can be observed. An anticrossing can be seen when the optical mode is tuned through interlayer excitonic energy. Because the shift of modified Mie resonant mode is limited due to the influence of cavity, the anticrossing behavior is incomplete compared with other cases.

To further analysis the origins of the scattering line shape, the coupled oscillators model was used to fit the spectral splitting. The scattering cross section is given by^39^

(1) $$\sigma_{\mathrm{sca}}\left(\omega \right)\;\;\propto \;\;\omega^{4}\left|\frac{\left(\omega^{2}\;\;-\;\;\omega_{\mathrm{ex}}^{2}\;\;+\;\;i\omega \gamma_{\mathrm{ex}}\right)}{\left(\omega^{2}\;\;-\;\;\omega_{\mathrm{MD}}^{2}\;\;+\;\;i\gamma_{\mathrm{MD}}\omega \right)\left(\omega^{2}\;\;-\;\;\omega_{\mathrm{ex}}^{2}\;\;+\;\;i\gamma_{\mathrm{ex}}\omega \right)\;\;-\;\;4\omega^{2}g^{2}}\right|$$

where *g* is the coupling strength, ω_ex_ and ω_MD_ are the energies of interlayer exciton and enhanced MD mode, and γ_ex_ and γ_MD_ are the dissipation rates (linewidths) of excitonic resonances and the uncoupled enhanced MD mode. Based on the strong coupling theory,^39^ the criterion 2*g* ≥ (γ_MD_ − γ_ex_)/2 should be satisfied to realize mode splitting. While Fano resonances would happen when 2*g* ≤ (γ_MD_ − γ_ex_)/2. In our case, The dissipation rates are γ_MD_ ≈ 270 meV and γ_ex_ ≈ 75 meV. Therefore, *g* can be calculated to 58 meV through fitting process. That means strong coupling and mode splitting can be realized in our case. Considering the enhanced MD modes are hybrid mode and have no linear shift with sizes, the mode splitting parameters varies with sizes. So, here, we used the Si NP with a diameter of 170 nm as an example to calculate the coupling strength. What should be noticed is that the absorption and emission peaks of TMDs locate at different wavelengths. Compared with excitonic peaks in PL spectra, absorption and scattering peaks in both WS_2_ and MoS_2_ blue shift.^24^, ^40^ Therefore, the scattering dip at 2.00 eV in Figure 3d still locates between the absorption peaks of WS_2_ and MoS_2_, which indicates it is interlayer process.

After studying the strong coupling in scattering spectrum, one can go back to understand the tuning of PL emission in Figure 2. The relationship between exciton energy and the diameters of NPs is plotted in Figure 3e. The variation of normal ED and MD modes and the modified MD mode are also added in Figure 3e. The deviation from initial interlayer exciton energy is observed in both coherent stack and random stack samples, and the final energy seems to converge to the same value. Without Si NPs, the interlayer emission peaks locate between the peaks from pure WS_2_ and MoS_2_ (Figure 1f). After adding Si NPs, the tunable control of interlayer excitons drives their energies further away from the location of pure WS_2_ and MoS_2_ excitons. For coherent‐stacking heterostructure, red‐shift of exciton emission when the size of NP is lower than 110 nm arises from the thermal and strain effects.^32^ Further increasing the sizes of NP enhances the optical resonant mode which becomes dominated from the competitive mechanism. Those effects in random‐stacking sample are much weaker because of much stronger PL intensity and weaker interlayer coupling which is stable when surrounding changes. In general, the red‐shift in random‐stacking heterostructure and the blue‐shift in coherent‐stacking heterostructure result from the strong coupling with cavity enhanced and broadened MD mode. The enhanced MD mode is stronger and more overlapped with excitonic emission when the sizes of NPs are larger, therefore the tuning effect is more obvious when increasing sizes. Strong coupling can be observed through both scattering and PL spectra. Some works have analyzed the correlation between scattering and PL and found the strong coupling in PL is more complex.^41^, ^42^ Some weakly absorbing dark states may contribute to the PL, while some bright modes in reflection or absorption may be greatly suppressed in PL due to the interference with optical resonant modes. In these systems, only lower polariton (LP) branch was observed while upper polariton (UP) is inactive due to fast nonradiative energy transfer to uncoupled incoherent states. Similarly, in our case, only one branch was observed in PL spectra and usual mode splitting changed to the shift of one branch.

Evolution of cavity enhanced MD mode is further described via near‐field profiles in Figure 3f. Here Si NP with a diameter of 170 nm is chosen as an example. Both electric and magnetic fields reach peak values at four photon energies: 1.45, 1.78, 2.03, and 2.36 eV. As a comparison, the near field profiles of Si NPs on pure silicon oxide are also presented in Figure 3f, which shows the feature of conventional Mie resonators. The ED mode for the Si NP on SiO_2_ is located around 2.36 eV, while the electric field enhancement of the Si NP on cavity at the same photon energy is even higher. Stronger electric field enhancements from Si NP on cavity not only occur at the ED mode but also exist at 1.45, 1.78, and 2.03 eV. Furthermore, a large portion of electric fields spread out and distribute on surface and at the interfaces with 2D materials because of the existence of cavity. This broader, stronger and extended electric mode is beneficial to realize strong coupling. For magnetic field distributions, Si NPs on cavity also possess stronger enhancement than Si NPs on SiO_2_ except the field profile at 1.78 eV, where the MD mode happens in normal Mie resonators. This phenomenon further indicates the conventional MD mode (displacement current loop) is partially break up and re‐engineered as a quasi‐surface mode. In Figure S4 (Supporting Information), we simulated the field distributions of a 140 nm Si NP on cavity and on SiO_2_ at 1.45, 1.78, 2.15, and 2.43 eV. In general, both electric and magnetic field enhancements in Si NPs on cavity are stronger than in normal Si NPs. Because smaller size compared with the 170 nm Si NP described in Figure 3, the modified mode is narrower. In the range from 1.78 to 2.15 eV, the behaviors of field distributions are different. When the photon energy equals 1.78 eV, both electric and magnetic field enhancements are stronger in 170 nm Si NP than in 140 nm Si NP because MD mode cannot be generated in 140 nm Si NP (see Figure 3c). While the field enhancement factors are comparable in Si NPs with two sizes at 2.15 eV because the MD mode in 140 nm Si NP comes into effects. Similarly, for smaller Si NPs, MD and ED modes further blue shift and no Mie resonant modes can be generated in the range from 1.78 to 2.15 eV, therefore the electric and magnetic fields become much weaker. The variation trend of enhance MD mode is plotted in Figure 3e to help one understand why larger Si NPs bring stronger coupling.

**Figure 4:**
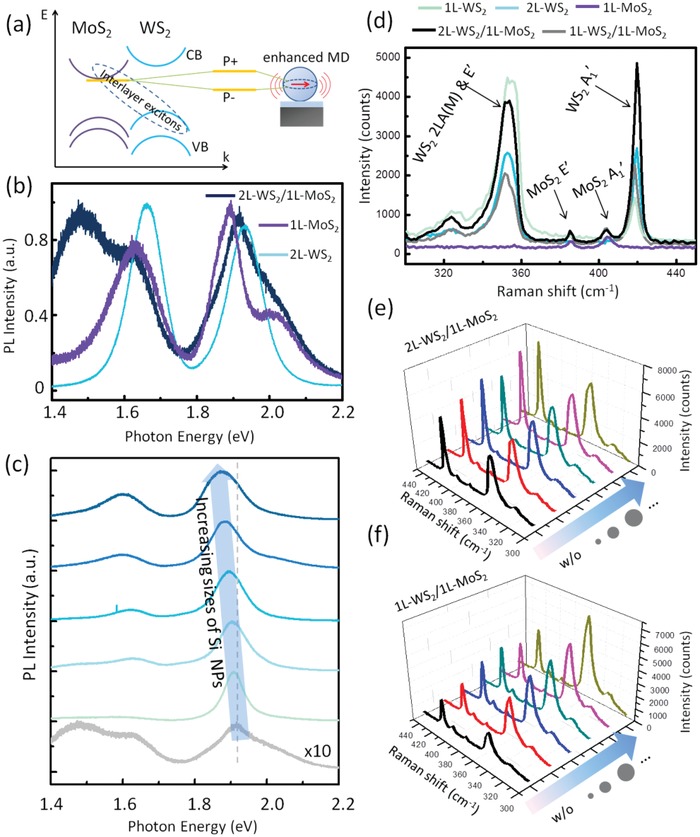
Detailed study on strong coupling. a) Schematic showing the strong coupling and mode splitting. b) PL spectra of WS_2_ bilayer (blue curve), MoS_2_ monolayer (purple curve), and the WS_2_/MoS_2_ heterostructure (black curve) on designed SiO_2_/Si substrate. c) Tunable PL spectra of WS_2_/MoS_2_ area with different Si NPs on. Curves with darker colors mean Si NPs with larger sizes. d) Raman spectra of monolayers, the bilayer, and heterostructures. Raman spectra of the e) 2L WS_2_/1L MoS_2_ heterostructure and f) 1L WS_2_/1L MoS_2_ heterostructure without Si NP (black curve) and with Si NPs with increasing sizes.

The strong coupling between enhanced MD mode in the Si NP on cavity and the interlayer exciton in the WS_2_/MoS_2_ heterostructure induces the Rabi splitting in scattering spectra and the spectral tuning of excitonic emission as the schematic shown in **Figure** **4**a. The coupling strength *g* in hybrid system can be described by^28^

(2) $$g\;\;\propto \;\;N^{\frac{1}{2}}\mu_{J}\left|E_{\mathrm{vac}}\right|\;\;=\;\;\mu_{J}\sqrt{\frac{n_{x}\hbar \omega }{2\varepsilon \varepsilon_{0}}}\sqrt{\frac{V_{x}}{V}}$$

where μ_J_ is the excitonic transition dipole moment, *n*_x_ is the density of excitons, *V*_x_ is the result of the overlap integral between the resonant mode and the surrounding excitons at the interface and *V* is the volume of resonant mode. Obviously, larger coupling strength can be obtained through increasing the effective mode volume or increasing the excitonic transition dipole movement. In our case, the enhanced quasi‐surface Mie resonant mode boosts the overlap between light field and excitons. Furthermore, since the thickness of WS_2_/MoS_2_ heterostructure is doubled compared with monolayers, the overlap and interaction region is larger. On the other hand, the transition dipole moment can be calculated as $\mu_{J}{}^{2}\;\;=\;\;{\left(\frac{eh}{2E_{0}}\right)}^{2}\frac{E_{g}}{m_{c}}$, where *E*_0_ is the transition energy of the exciton, *E*_g_ is the bandgap, and *m*_c_ is the effective mass of the conduction electron.^43^ Therefore, μ_J_ of interlayer excitons has a similar value compared with pure WS_2_ and MoS_2_. Considering μ_J_ is proportional to the effective dielectric constant,^31^ interlayer excitons located between WS_2_ and MoS_2_ monolayers and placed into the cavity have a surrounding media with large dielectric function, which is beneficial to realize strong coupling. The coupling strength g also depends on the orientation of μ_J_ as $g_{i}\;\;=\;\;|{\vec{\mu }}_{J}||{\vec{E}}_{\mathrm{vac}}(r_{i})|\cos\theta_{i}$, where θ_i_ is the angle between μ_J_ and the coupling electric field. Since the out‐of‐plane interlayer dipole is perpendicular to the substrate and in the same direction as the cavity enhanced resonant modes, this platform can produce larger *g* than in‐plane excitons in WS_2_ or MoS_2_ monolayers.^15^, ^29^ All these factors discussed above make interlayer excitons coupling with enhanced Mie resonances reach the criterion for strong coupling. Although previous works have studied the coupling between single Si NPs and monolayer TMDs, either Purcell effect^32^ or unobvious Rabi splitting^31^, ^39^ was observed, which indicates the coupling is still weak.

Other types of heterostructure were also studied as shown in Figure 4b,c. The vdW heterostructure containing bilayer WS_2_ and monolayer MoS_2_ in Figure 2d was measured. Figure 4b indicates the generation of interlayer exciton located between the direct band transition energy of MoS_2_ and WS_2_. Besides a hybrid peak from the indrect band transition of bilayer WS_2_ and the cavity mode, the other interlayer excitonic emission is also observed at 1.50 eV. Figure 4c is the PL spectra of three‐layer heterostructure with Si NPs with different sizes. Besides the significant enhancement after putting Si NPs on, the gradual red shift (maximum is 0.04 eV) of interlayer excitons is also observed when increasing the diameter of Si NPs. The other interlayer excitonic state at 1.50 eV disappears with Si NPs and cannot be investigated because Si NPs on cavity only amplify the excitons around 1.90 eV.

The Raman spectra of monolayers and heterostructures are presented in Figure 4d. Four peaks at 357, 385, 406, and 419 cm^−1^ correspond to the E′ and A_1_′ modes of MoS_2_ and WS_2_.^10^ Comparing with pure monolayer WS_2_, the peaks of bilayer WS_2_ and monolayer WS_2_ on MoS_2_ all slightly shift and change ratio, which demonstrates the influence of MoS_2_. After depositing Si NPs on heterostructures, Raman spectra also change as shown in Figure 4e,f. Raman peaks in the 2L WS_2_/1L MoS_2_ heterostructure slightly increase, while the peaks in 1L WS_2_/1L MoS_2_ heterostructure strongly enhance especially the E′ peak of WS_2_. The enhancement effect arises from the near‐field enhancement at excitation wavelength to enhance light‐trapping and absorption. Larger Si NPs lead to larger Raman enhancement, which further demonstrates better performance of field enhancement in larger Si NPs.

## 3 Conclusion

In conclusion, using Si NPs on the cavity as a platform, we first observe the tuning on interlayer exciton transition emission based on the size of Si NPs. Significant red shift (0.03 eV) and blue shift (0.05 eV) have been observed in the random stack and coherent stack heterostructures, respectively. Rabi splitting in the scattering spectrum has also been observed in heterostructures with Si NPs decorated. A detailed analysis shows how the unique cavity‐enhanced Mie resonant mode affects the properties of interlayer excitons. This tuning mechanism can also expand to a three‐layer heterostructure (2L WS_2_/1L MoS_2_). Our findings bridge the gap on understanding interlayer excitons and strong coupling in fundamental studies and show promising applications of vdW heterostructures in nanophotonics.

## 4 Experimental Section

*Fabrication of WS_2_/MoS_2_ Heterostructures*: First, 50 nm high quality SiO_2_ insulator layer was deposited on purchased SOI substrates (Si 200 nm+SiO_2_ 375 nm+bottom Si 525 μm) using Inductively Coupled Plasma Chemical Vapor Deposition (ICPCVD). Second, the WS_2_ flakes obtained from mechanically exfoliation of the bulk crystal with Nitto tape were transferred to the viscoelastic polydimethylsiloxane (PDMS) substrate. Third, the PDMS transparent film was pressed to attach the substrate and peeled off slowly. Fourth, all‐dry transfer^33^ was used to place MoS_2_ flakes on the wanted WS_2_ few layers. Finally, the Si NPs with diameters from 100 to 200 nm were fabricated by the femtosecond laser ablation in liquid (fs‐LAL).^44^ Then, one drop of the silicon colloid was transferred onto the substrate. During the evaporation process, Si NPs have a relative high probability to be placed on WS_2_/MoS_2_ heterostructures.

*Optical Characterization*: PL spectra and Raman spectra were measured by a Renishaw inVia confocal Raman microscope using a 514 nm and a 50 × objective lens (NA = 0.75) at room temperature. The laser spot size is less than 850 nm,^45^ which is small enough to embody the interaction between Si NPs and 2D materials. The dark‐field scattering spectra of Si NPs on heterostructures were measured by an optical microscope (BX51, Olympus) integrated with a spectrograph (IsoPlane 160, Princeton Instruments). The oblique incident white light with a 53° incident angle was illuminated on the Si NPs, and the scattered light was collected by the same objective. The precise location in optical measurement was determined by matching “black dots” in the bright field optical image and “bright dots” in the dark field optical image with different Si NPs in SEM images.

*Numerical Simulations*: The scattering spectra and near‐field distributions were calculated using FDTD method (Lumerical Solution, Inc). Unpolarized incident total‐field scattered‐field (TFSF) light source was used with the wavelength range from 300 to 900 nm (4.13 to 1.38 eV). A mesh size of 1 nm was used for the illuminated region, and the dielectric function of silicon was obtained from the widely used Palik's parameters.^46^ The diameters of Si NPs were set according to the experimental measurement from SEM images.

## Conflict of Interest

The authors declare no conflict of interest.

## Supporting information

Supplementary

## References

[1] Q. H. Wang , K. Kalantar‐Zadeh , A. Kis , J. N. Coleman , M. S. Strano , Nat. Nanotechnol. 2012, 7, 699. 23132225 10.1038/nnano.2012.193

[2] K. F. Mak , C. Lee , J. Hone , J. Shan , T. F. Heinz , Phys. Rev. Lett. 2010, 105, 136805. 21230799 10.1103/PhysRevLett.105.136805

[3] S. Mouri , Y. Miyauchi , K. Matsuda , Nano Lett. 2013, 13, 5944. 24215567 10.1021/nl403036h

[4] Y. Lin , X. Ling , L. Yu , S. Huang , A. L. Hsu , Y.‐H. Lee , J. Kong , M. S. Dresselhaus , T. Palacios , Nano Lett. 2014, 14, 5569. 25216267 10.1021/nl501988y

[5] J. Shang , X. Shen , C. Cong , N. Peimyoo , B. Cao , M. Eginligil , T. Yu , ACS Nano 2015, 9, 647. 25560634 10.1021/nn5059908

[6] Z. Li , T. Wang , Z. Lu , C. Jin , Y. Chen , Y. Meng , Z. Lian , T. Taniguchi , K. Watanabe , S. Zhang , D. Smirnov , S.‐F. Shi , Nat. Commun. 2018, 9, 3719. 30213927 10.1038/s41467-018-05863-5 PMC6137082

[7] M. S. Kim , S. J. Yun , Y. Lee , C. Seo , G. H. Han , K. K. Kim , Y. H. Lee , J. Kim , ACS Nano 2016, 10, 2399. 26758415 10.1021/acsnano.5b07214

[8] Y. Wang , C. Cong , W. Yang , J. Shang , N. Peimyoo , Y. Chen , J. Kang , J. Wang , W. Huang , T. Yu , Nano Res. 2015, 8, 2562.

[9] D. Unuchek , A. Ciarrocchi , A. Avsar , K. Watanabe , T. Taniguchi , A. Kis , Nature 2018, 560, 340. 30046107 10.1038/s41586-018-0357-y

[10] Y. Gong , J. Lin , X. Wang , G. Shi , S. Lei , Z. Lin , X. Zou , G. Ye , R. Vajtai , B. I. Yakobson , H. Terrones , M. Terrones , B. K. Tay , J. Lou , S. T. Pantelides , Z. Liu , W. Zhou , Nat. Mater. 2014, 13, 1135. 25262094 10.1038/nmat4091

[11] E. Torun , H. P. C. Miranda , A. Molina‐Sanchez , L. Wirtz , Phys. Rev. B 2018, 97, 245427.

[12] C. Jin , J. Kim , M. I. B. Utama , E. C. Regan , H. Kleemann , H. Cai , Y. Shen , M. J. Shinner , A. Sengupta , K. Watanabe , T. Taniguchi , S. Tongay , A. Zettl , F. Wang , Science 2018, 360, 893. 29798880 10.1126/science.aao3503

[13] H. Heo , J. H. Sung , S. Cha , B.‐G. Jang , J.‐Y. Kim , G. Jin , D. Lee , J.‐H. Ahn , M.‐J. Lee , J. H. Shim , H. Choi , M.‐H. Jo , Nat. Commun. 2015, 6, 7372. 26099952 10.1038/ncomms8372 PMC4557351

[14] P. K. Nayak , Y. Horbatenko , S. Ahn , G. Kim , J.‐U. Lee , K. Y. Ma , A. R. Jang , H. Lim , D. Kim , S. Ryu , H. Cheong , N. Park , H. S. Shin , ACS Nano 2017, 11, 4041. 28363013 10.1021/acsnano.7b00640

[15] B. Miller , A. Steinhoff , B. Pano , J. Klein , F. Jahnke , A. Holleitner , U. Wurstbauer , Nano Lett. 2017, 17, 5229. 28742367 10.1021/acs.nanolett.7b01304

[16] J. Zhang , J. Wang , P. Chen , Y. Sun , S. Wu , Z. Jia , X. Lu , H. Yu , W. Chen , J. Zhu , G. Xie , R. Yang , D. Shi , X. Xu , J. Xiang , K. Liu , G. Zhang , Adv. Mater. 2016, 28, 1950. 26708256 10.1002/adma.201504631

[17] Y. Yu , S. Hu , L. Su , L. Huang , Y. Liu , Z. Jin , A. A. Purezky , D. B. Geohegan , K. K. Kim , Y. Zhang , L. Cao , Nano Lett. 2015, 15, 486. 25469768 10.1021/nl5038177

[18] M. Okada , A. Kutana , Y. Kureishi , Y. Kobayashi , Y. Saito , T. Saito , K. Watanabe , T. Taniguchi , S. Gupta , Y. Miyata , B. I. Yakobson , H. Shinohara , R. Kitaura , ACS Nano 2018, 12, 2498. 29481065 10.1021/acsnano.7b08253

[19] K.‐D. Park , T. Jiang , G. Clark , X. Xu , M. B. Raschke , Nat. Nanotechnol. 2018, 13, 59. 29158602 10.1038/s41565-017-0003-0

[20] S. Wang , S. Li , T. Chervy , A. Shalabney , S. Azzini , E. Origiu , J. A. Hutchison , C. Genet , P. Samori , T. W. Ebbesen , Nano Lett. 2016, 16, 4368. 27266674 10.1021/acs.nanolett.6b01475

[21] X. Zhang , S. Choi , D. Wang , C. H. Naylor , A. T. Charlie Johnson , E. Cubukcu , Nano Lett. 2017, 17, 6715. 28991494 10.1021/acs.nanolett.7b02777

[22] L. Zhang , R. Gogna , W. Burg , E. Tutuc , H. Deng , Nat. Commun. 2018, 9, 713. 29459736 10.1038/s41467-018-03188-x PMC5818602

[23] K. F. Mak , J. Shan , Nat. Photonics 2016, 10, 216.

[24] A. Krasnok , S. Lepeshov , A. Alu , Opt. Express 2018, 26, 15972. 30114850 10.1364/OE.26.015972

[25] D. G. Baranov , M. Wersall , J. Cuadra , T. J. Antosiewicz , T. Shegai , ACS Photonics. 2018, 5, 24.

[26] J. Yan , P. Liu , Z. Lin , H. Wang , H. Chen , C. Wang , G. Yang , ACS Nano 2015, 9, 2968. 25683067 10.1021/nn507148z

[27] P. Liu , J. Yan , C. Ma , Z. Lin , G. Yang , ACS Appl. Mater. Interfaces 2016, 8, 22468. 27502321 10.1021/acsami.6b05123

[28] J. Yan , C. Ma , P. Liu , C. Wang , G. Yang , Light: Sci. Appl. 2017, 6, e16197. 30167196 10.1038/lsa.2016.197 PMC6061887

[29] P. Rivera , J. R. Schaibley , A. M. Jones , J. S. Ross , S. Wu , G. Aivazian , P. Klement , K. Seyler , G. Clark , N. J. Ghimire , J. Yan , D. G. Mandrus , W. Yao , X. Xu , Nat. Commun. 2015, 6, 6242. 25708612 10.1038/ncomms7242

[30] M.‐E. Kleemann , R. Chikkaraddy , E. M. Alexeev , D. Kos , C. Carnegie , W. Deacon , A. C. de Pury , C. Grobe , B. de Nijs , J. Mertens , A. I. Tartakovskii , Nat. Commun. 2017, 8, 1296. 29101317 10.1038/s41467-017-01398-3 PMC5670138

[31] S. Lepeshov , M. Wang , A. Krasnok , O. Kotov , T. Zhang , H. Liu , T. Jiang , B. Korgel , M. Terrones , Y. Zheng , A. Alu , ACS Appl. Mater. Inferfaces 2018, 10, 16690. 10.1021/acsami.7b17112 29651843

[32] C. Ma , J. Yan , Y. Huang , G. Yang , Mater. Horiz. 2019, 6, 97.

[33] A. Castellanos‐Gomez , M. Buscema , R. Molenaar , V. Singh , L. Janssen , H. S. J. van der Zant , G. A. Steele , 2D Mater. 2014, 1, 011002.

[34] S. Tongay , W. Fan , J. Kang , J. Park , U. Koldemir , J. Suh , D. S. Narang , K. Liu , J. Ji , J. Li , R. Sinclair , J. Wu , Nano Lett. 2014, 14, 3185. 24845201 10.1021/nl500515q

[35] H.‐C. Kim , H. Kim , J.‐U. Lee , H.‐B. Lee , D.‐H. Choi , J.‐H. Lee , W. H. Lee , S. H. Jhang , B. H. Park , H. Cheong , S.‐W. Lee , H.‐J. Chung , ACS Nano 2015, 9, 6854. 26143940 10.1021/acsnano.5b01727

[36] N. Scheuschner , O. Ochedowski , A.‐M. Kaulitz , R. Gillen , M. Schleberger , J. Maultzsch , Phys. Rev. B 2014, 89, 125406.

[37] H. Y. Jeong , U. J. Kim , H. Kim , G. H. Han , H. Lee , M. S. Kim , Y. Jin , T. H. Ly , S. Y. Lee , Y.‐G. Roh , W.‐J. Joo , S. W. Hwang , Y. Park , Y. H. Lee , ACS Nano 2016, 10, 8192. 27556640 10.1021/acsnano.6b03237

[38] M. Buscema , G. A. Steele , H. S. J. van der Zant , A. Castellanos‐Gomez , Nano Res. 2014, 7, 561.

[39] H. Wang , J. Wen , W. Wang , N. Xu , P. Liu , J. Yan , H. Chen , S. Deng , ACS Nano 2019, 13, 1739. 30629416 10.1021/acsnano.8b07826

[40] K. F. Mak , K. He , C. Lee , G. H. Lee , J. Hone , T. F. Heinz , J. Shan , Nat. Mater. 2013, 12, 207. 23202371 10.1038/nmat3505

[41] J. R. Tischler , M. S. Bradley , V. Bulovic , J. H. Song , A. Nurmikko , Phys. Rev. Lett. 2005, 95, 036401. 16090759 10.1103/PhysRevLett.95.036401

[42] M. Wersall , J. Cuadra , T. J. Antosiewicz , S. Balci , T. Shegai , Nano Lett. 2017, 17, 551. 28005384 10.1021/acs.nanolett.6b04659

[43] E. J. Sie , J. W. Mclver , Y.‐H. Lee , L. Fu , J. Kong , N. Gedik , Nat. Mater. 2015, 14, 290. 25502098 10.1038/nmat4156

[44] J. Yan , C. Ma , P. Liu , C. Wang , G. Yang , Nano Lett. 2017, 17, 4793. 28686459 10.1021/acs.nanolett.7b01566

[45] J. Yan , C. Ma , P. Liu , G. Yang , ACS Photonics 2017, 4, 1092.

[46] E. D. Palik , Handbook of Optical Constants of Solids, Academic Press, Cambridge, Massachusetts, USA 1998.

