# Supplementary material for: Tunable Control of Interlayer Excitons in WS2/MoS2 Heterostructures via Strong Coupling with Enhanced Mie Resonances
Source: Adv Sci (Weinh). 2019 Apr 2;6(11):1802092. doi: 10.1002/advs.201802092 (PMC6548949; doi:10.1002/advs.201802092)
Supplement: Supplementary file 1 — Supplementary [file ADVS-6-1802092-s001.pdf]

Copyright WILEY-VCH Verlag GmbH & Co. KGaA, 69469 Weinheim, Germany, 2019.

ADVANCED SCIENCE
Open Access

Supporting Information

for *Adv. Sci.,* DOI: 10.1002/advs.201802092

Tunable Control of Interlayer Excitons in WS_2/MoS_2 Heterostructures via Strong Coupling with Enhanced Mie Resonances

*Jiahao Yan, Churong Ma, Yingcong Huang, and Guowei Yang**

Supporting Information (SI) for "Tunable control of interlayer excitons in WS_2/MoS_2 heterostructures via strong coupling with enhanced Mie resonances"

Jiahao Yan, Churong Ma, Yingcong Huang & Guowei Yang*

State Key Laboratory of Optoelectronic Materials and Technologies, Nanotechnology Research Center, School of Materials Science & Engineering, Sun Yat-sen University, Guangzhou 510275, Guangdong, P. R. China

*Corresponding author: stsygw@mail.sysu.edu.cn

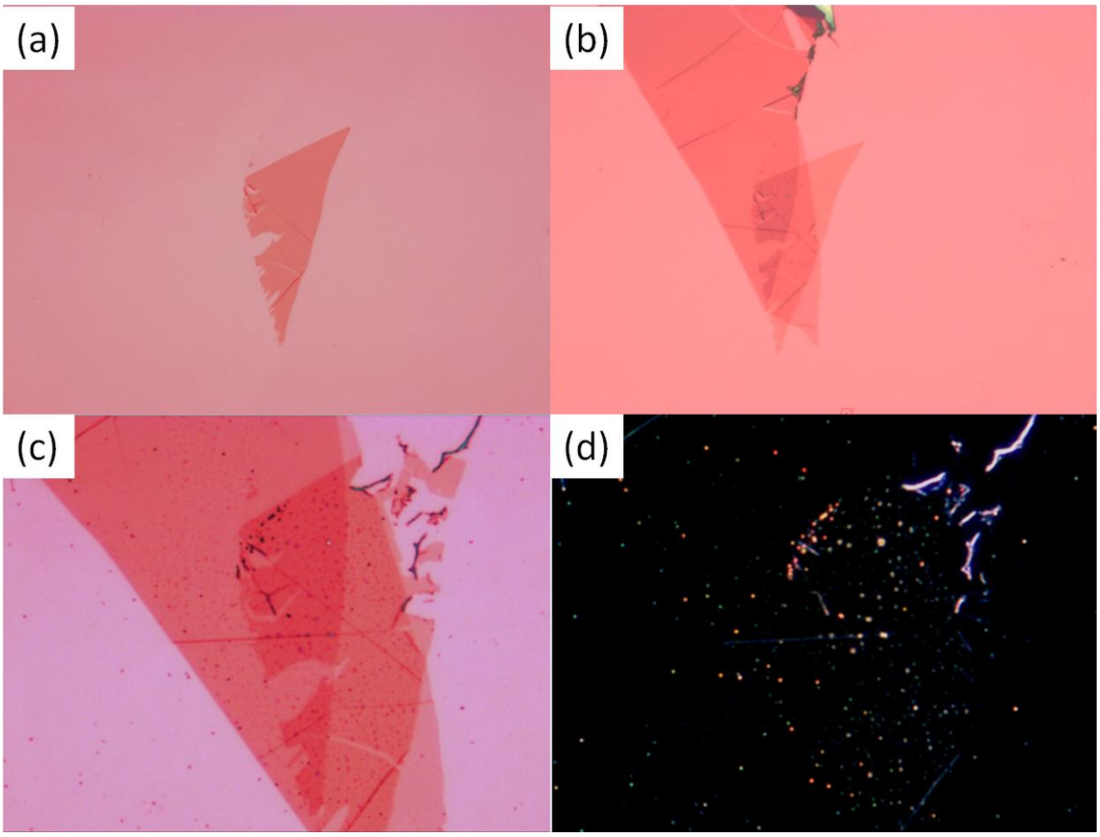

Figure S1. (a) The optical image of a MoS_2 monolayer. (b) The optical image of the fabricated random stack WS_2/MoS_2 heterostructure. (c)(d) Bright field (c) and dark field (d) optical images of the heterostructure after depositing Si NPs.

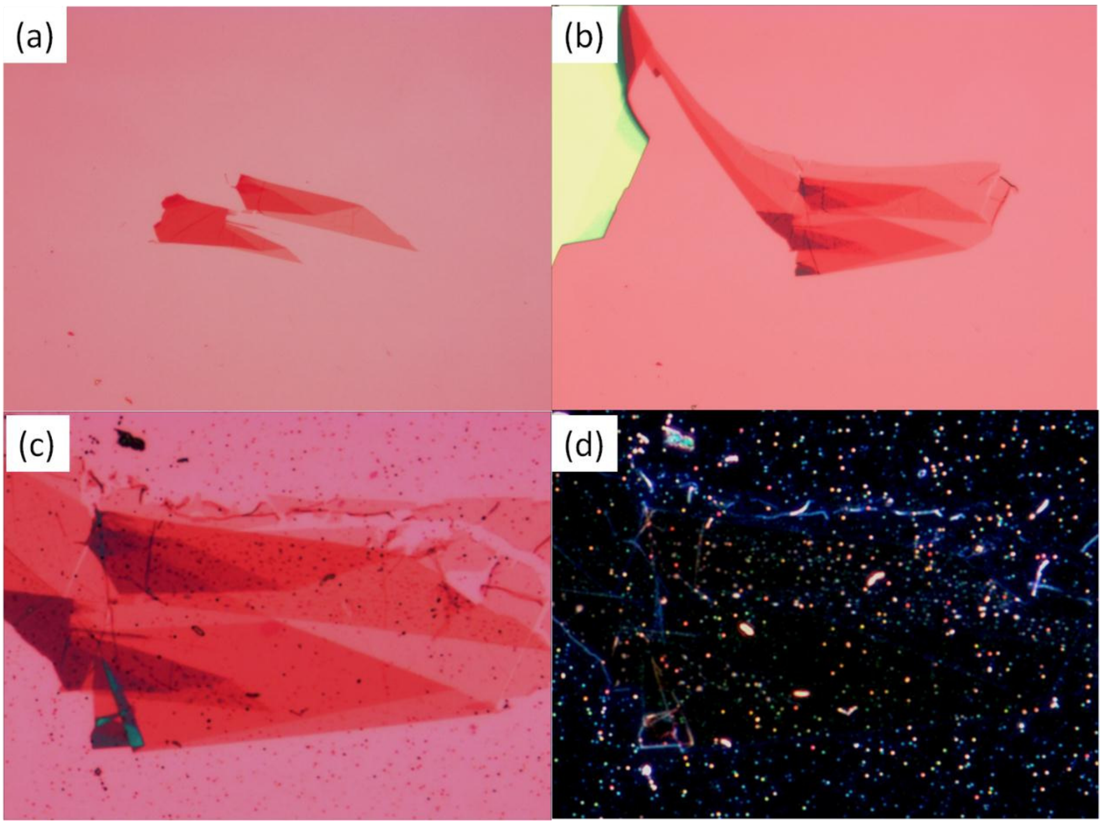

Figure S2. (a) The optical image of a MoS_2 monolayer. (b) The optical image of the fabricated coherent stack WS_2/MoS_2 heterostructure. (c)(d) Bright field (c) and dark field (d) optical images of the heterostructure after depositing Si NPs.

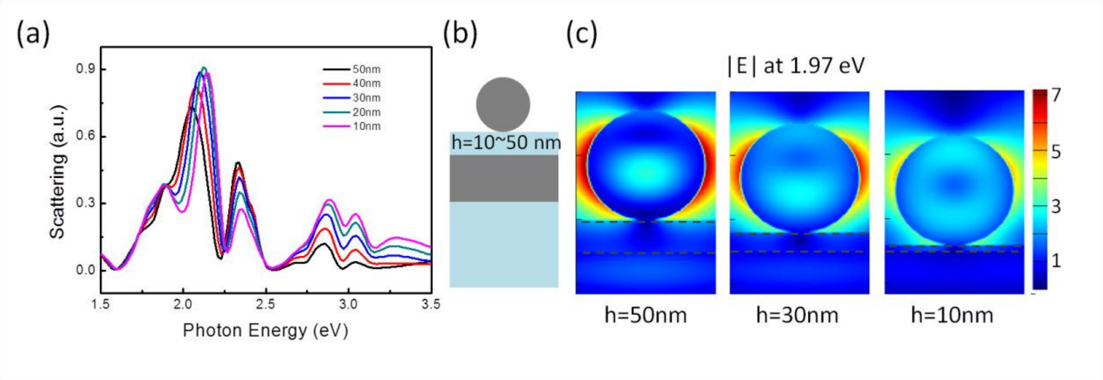

Figure S3. (a) Simulated scattering spectra of a Si NP with diameter of 170 nm on designed SiO_2/Si substrate. The thickness of top oxide layer changes from 50 nm (our design) to 10 nm. (b) Schematic of the designed structure. (c) Electric field distributions of Si NPs on oxide layer with different thicknesses (50, 30, 10 nm) at 1.97 eV.

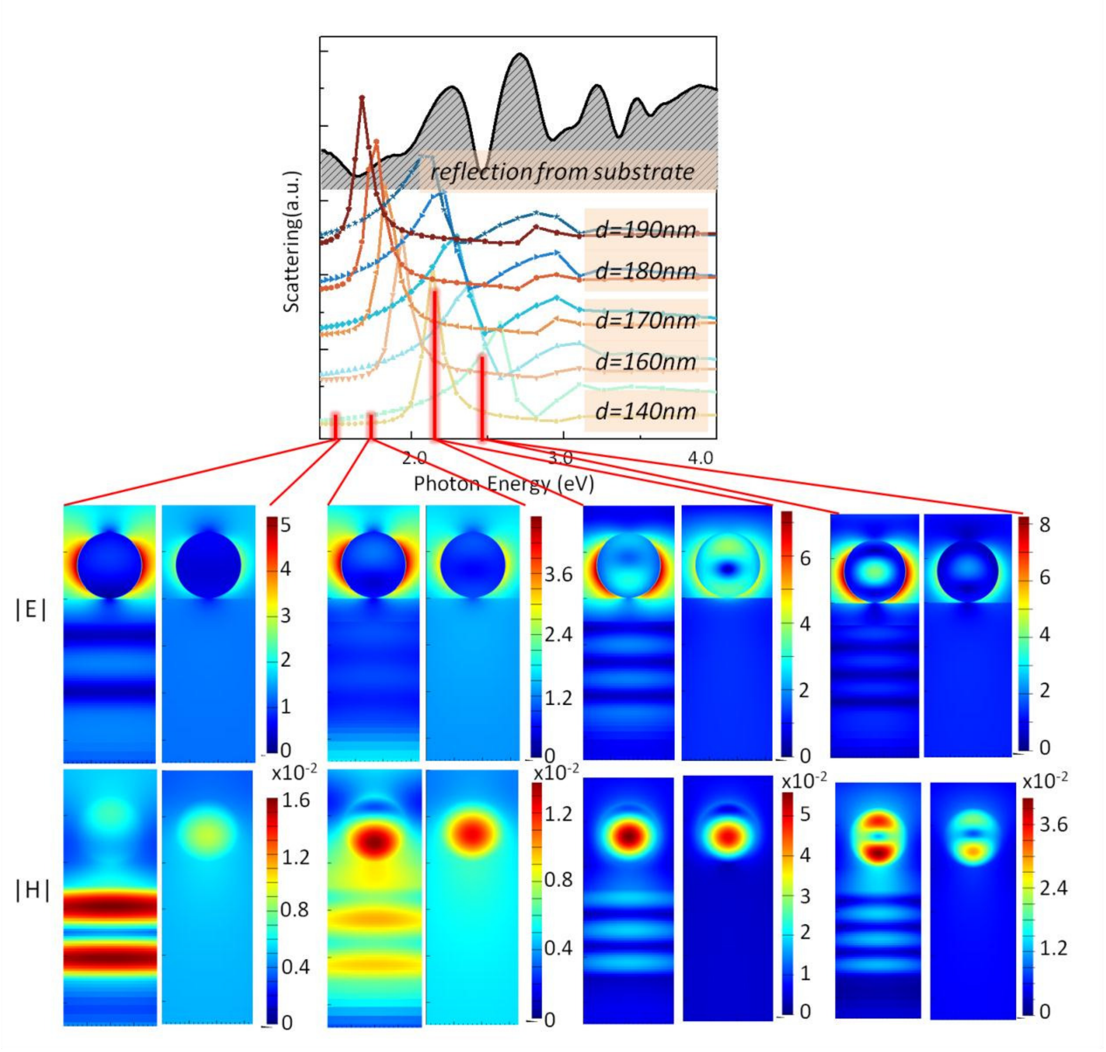

Figure S4. Calculated scattering contributed by ED and MD modes with the simulated reflection from designed SiO_2/Si substrate. ED modes are plotted using series of cool colors, while MD modes are plotted using warm colors. Electric (|E|) and magnetic (|H|) field distributions of Si NPs on cavity and on pure oxide at four different locations in spectrum marked by red bars.
